# Bilateral subconjunctival hemorrhage secondary to abciximab use: case report

**DOI:** 10.1590/1516-3180.2017.0182150717

**Published:** 2017-12-18

**Authors:** Selim Kul, Muhammet Raşit Sayın

**Affiliations:** I MD. Cardiology Specialist, Saglik Bilimleri University, Department of Cardiology, Trabzon Ahi Evren Cardiovascular and Thoracic Surgery Research and Application Center, Saglik Bilimleri University, Trabzon, Turkey.; II MD. Associate Professor, Saglik Bilimleri University, Department of Cardiology, Trabzon Ahi Evren Cardiovascular and Thoracic Surgery Research and Application Center, Saglik Bilimleri University, Trabzon, Turkey.

**Keywords:** Conjunctivitis, acute hemorrhagic, Abciximab [supplementary concept], Coronary disease

## Abstract

**CONTEXT:**

There are no reports on cases of subconjunctival hemorrhage due to use of glycoprotein IIb/IIIa inhibitors. In this report, we present the case of a patient with bilateral subconjunctival hemorrhage after receiving abciximab.

**CASE REPORT:**

A 40-year-old male patient underwent coronary angiography after acute anterior myocardial infarction and a coronary stent was placed. Abciximab was added to the therapy because of stent thrombosis. Bilateral subconjunctival hemorrhage was observed after the administration of the abciximab treatment. We treated our patient by stopping abciximab and administering artificial tears.

**CONCLUSİON:**

For the first time in the literature, we presented the case of a patient with bilateral subconjunctival hemorrhage after receiving abciximab, which was managed conservatively.

## INTRODUCTION

Abciximab, a Fab fragment of the chimeric human-murine monoclonal antibody 7E3, interferes with platelet aggregation by binding to the glycoprotein IIb/IIIa receptors of platelets.^1^ Although no randomized trial has tested the use of abciximab as a bailout therapy in cases of ST-elevated myocardial infarction (STEMI), it has been found to be beneficial in cases of large intraluminal thrombus, slow reflow or no reflow, and in relation to other thrombotic complications during angiography.^2^

The major side effect of abciximab is bleeding. The EPIC trial included patients undergoing high-risk angioplasty procedures, among whom 14% suffered major bleeding after receiving a bolus followed by infusion of abciximab, compared with 7% of the patients receiving placebo.^3^

For the first time in the literature (Table 1), we present the case of a patient with bilateral subconjunctival hemorrhage after receiving abciximab.

**Table 1:** Strategies used for search in electronic databases on June 11, 2017

Database	Search strategy	References retrieved
MEDLINE (via PubMed)	(“Conjunctivitis, Acute Hemorrhagic”[Mesh]) AND (“abciximab” [Supplementary Concept])	0
Embase (via Elsevier)	(Abciximab OR Tirofiban OR Eptifibatide) AND (Subconjunctival Hemorrhage)	0

## CASE REPORT

A 40-year-old man was brought to the emergency department of our hospital with a history of chest pain for the last two hours. His past history was notable only for smoking as a cardiovascular risk factor. His vital signs included arterial blood pressure of 120/80 mmHg and a pulse rate of 66 bpm. His electrocardiogram was consistent with ST elevation in leads V1 to V6. His echocardiogram was notable for anterior and apical hypokinesia, with an ejection fraction of 40%. All his biochemical and blood count parameters were within normal limits.

**Figure 1:**
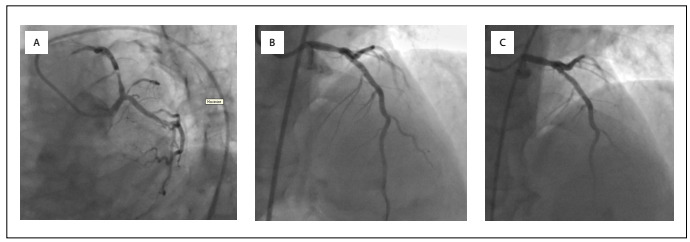
A. Coronary angiogram showing 99% stenosis in the proximal portion of the left anterior descending artery (LAD). B. A 3.0 mm x 24 mm drug-eluting stent was placed at a pressure of 12 atm in the severely stenotic LAD segment, without predilatation. C. Coronary angiography showing thrombus in LAD stent.

The patient was diagnosed as presenting anterior myocardial infarction and was transferred to the catheter laboratory for primary percutaneous coronary intervention (PCI). He was administered loading doses of 600 mg, 300 mg and 8000 U of clopidogrel, acetylsalicylic acid and unfractionated heparin, respectively. A coronary angiogram showed 99% stenosis in the proximal portion of the left anterior descending artery (LAD) (Figure 1A). The left circumflex artery was free of any stenosis and the right coronary artery had a 30% non-significant lesion in its mid-segment. A 3.0 mm x 24 mm drug-eluting stent was placed at a pressure of 12 atm in the severely stenotic LAD segment, without predilatation (Figure 1B).

The patient was then transferred to the coronary care unit for further observation. Thirty minutes later, the patient was defibrillated at 270 J due to an episode of ventricular fibrillation. Because his chest pain intensified, he was taken back to the catheter laboratory for a check on stent patency. Coronary angiography showed a thrombus in the LAD stent (Figure 1C).

**Figure 2:**
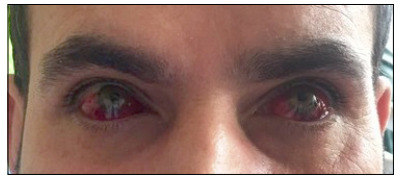
Bilateral subconjunctival hemorrhage secondary to use of abciximab.

A decision was made to administer abciximab to prevent further thrombosis inside the stent lumen or elsewhere in the coronary circulation. Abciximab was administered as a bolus at a dose of 0.25 mg/kg, followed by infusion at a dose of 0.125 mcg/kg/min intravenously. However, 20 minutes after the start of the infusion, the patient developed subconjunctival hemorrhage in both eyes (Figure 2). Therefore, infusion of the drug was stopped, but administration of acetylsalicylic acid and clopidogrel was continued.

An ophthalmology consultation was obtained for the patient, and it was recommended that he should receive conservative treatment consisting of artificial tears. The chest pain did not recur in the coronary intensive care unit after administration of abciximab was stopped. He was transferred to the cardiology ward, and a few days later, a coronary angiogram showed absence of thrombus and the patient was discharged.

He returned to the cardiology outpatient clinic for a follow-up visit, at which it was noted that his conjunctival hemorrhage had been completely eliminated. He was asymptomatic, while continuing to take acetylsalicylic acid and clopidogrel two months later.

## DISCUSSION

Anticoagulants and antiaggregant medications that prevent thrombosis and clot formation are central to management of patients undergoing percutaneous coronary intervention. Nevertheless, the risk of bleeding is inevitably increased through use of powerful antiplatelet and anticoagulant agents. Subconjunctival hemorrhage is one of the most common eye disorders, especially among individuals over the age of 50 years. Hypertension is one of the major risk factors of this condition. Anticoagulants in the form of low-dose heparin and warfarin have been linked to subconjunctival hemorrhage at an incidence of 1.5% to 5%.^4^^,^^5^ Dabigatran, a novel direct thrombin inhibitor with anticoagulant properties, has been reported to cause subconjunctival hemorrhage.^6^

Our patient suffered bilateral subconjunctival hemorrhage. So far, no study has reported such an association for abciximab, tirofiban or eptifibatide. Our patient may have developed this complication due to abciximab, acetylsalicylic acid, clopidogrel or combined use of these three medications. We have not come across any report in the literature on a case of conjunctival hemorrhage due to use of clopidogrel. There has only been one case of conjunctival hemorrhage due to a high dose of aspirin.^7^ We used the usual doses of acetylsalicylic acid and clopidogrel. Since our patient’s subconjunctival hemorrhage regressed after abciximab was withdrawn, despite continuation of use of clopidogrel and acetylsalicylic acid during the follow-up, we consider that this complication was due solely to use of abciximab. Our patient suffered a rare bleeding complication due to abciximab, i.e. bilateral spontaneous subconjunctival hemorrhage. Even though subconjunctival hemorrhage may also occur as a result of rupture of small subconjunctival blood vessels, either idiopathically or after trauma or the Valsalva maneuver, our patient had neither of these causes.

Subconjunctival hemorrhage usually has a benign course and is self-limiting. It is minimally symptomatic and does not necessitate any specific therapy; and this is particularly true for patients who are not using anticoagulants.

## CONCLUSION

Bilateral subconjunctival hemorrhage due to use of abciximab has not been previously reported in the literature. Bilateral subconjunctival hemorrhage was seen after abciximab use in our case for the first time in the literature. Bilateral subconjunctival hemorrhage was a rare complication due to use of abciximab in our patient. We treated our patient by stopping his use of abciximab and administering artificial tears and followed him up conservatively on an outpatient basis. We achieved the outcome of complete spontaneous healing. In cases of subconjunctival hemorrhage due to abciximab use, abciximab treatment must be discontinued and full recovery can be achieved by artificial tearing and conservative treatment.

## References

[1] Ibbotson T, McGavin JK, Goa KL (2003). Abciximab: an updated review of its therapeutic use in patients with ischaemic heart disease undergoing percutaneous coronary revascularisation. Drugs.

[2] Windecker S, Kolh P, Alfonso F, Authors/Task Force members (2014). 2014 ESC/EACTS Guidelines on myocardial revascularization: The Task Force on Myocardial Revascularization of the European Society of Cardiology (ESC) and the European Association for Cardio-Thoracic Surgery (EACTS). Developed with the special contribution of the European Association of Percutaneous Cardiovascular Interventions (EAPCI). Eur Heart J.

[3] EPIC Investigators (1994). Use of a monoclonal antibody directed against the platelet glycoprotein IIb/IIIa receptor in high-risk coronary angioplasty. N Engl J Med.

[4] Schulman S, Kearon C, Kakkar AK (2009). Dabigatran versus warfarin in the treatment of acute venous thromboembolism. N Engl J Med.

[5] Baetz BE, Spinler SA (2008). Dabigatran etexilate: an oral direct thrombin inhibitor for prophylaxis and treatment of thromboembolic diseases. Pharmacotherapy.

[6] Nguyen TM, Phelan MP, Werdich XQ, Rychwalski PJ, Huff CM (2013). Subconjunctival hemorrhage in a patient on dabigatran (Pradaxa). Am J Emerg Med.

[7] Black RA, Bensinger RE (1982). Bilateral subconjunctival hemorrhage after acetylsalicylic acid overdose. Ann Ophthalmol.

